# Multi-Transduction-Mechanism Technology, an Emerging Approach to Enhance Sensor Performance

**DOI:** 10.3390/s23094457

**Published:** 2023-05-03

**Authors:** Youssef Ezzat Elnemr, Aya Abu-Libdeh, Gian Carlo Antony Raj, Yumna Birjis, Haleh Nazemi, Pavithra Munirathinam, Arezoo Emadi

**Affiliations:** Department of Electrical and Computer Engineering, University of Windsor, Windsor, ON N9B 3P4, Canada; elnemry@uwindsor.ca (Y.E.E.); abulibd1@uwindsor.ca (A.A.-L.); antonyrg@uwindsor.ca (G.C.A.R.); birjis@uwindsor.ca (Y.B.); nazemih@uwindsor.ca (H.N.); muniratp@uwindsor.ca (P.M.)

**Keywords:** capacitive, hybrid nanogenerators, microelectromechanical systems, multi-transduction, piezoelectric, electronic nose, sensor, transduction mechanisms, triboelectric, energy harvesting

## Abstract

Conventional sensor systems employ single-transduction technology where they respond to an input stimulus and transduce the measured parameter into a readable output signal. As such, the technology can only provide limited corresponding data of the detected parameters due to relying on a single transformed output signal for information acquisition. This limitation commonly results in the need for utilizing sensor array technology to detect targeted parameters in complex environments. Multi-transduction-mechanism technology, on the other hand, may combine more than one transduction mechanism into a single structure. By employing this technology, sensors can be designed to simultaneously distinguish between different input signals from complex environments for greater degrees of freedom. This allows a multi-parameter response, which results in an increased range of detection and improved signal-to-noise ratio. In addition, utilizing a multi-transduction-mechanism approach can achieve miniaturization by reducing the number of required sensors in an array, providing further miniaturization and enhanced performance. This paper introduces the concept of multi-transduction-mechanism technology by exploring different candidate combinations of fundamental transduction mechanisms such as piezoresistive, piezoelectric, triboelectric, capacitive, and inductive mechanisms.

## 1. Introduction

Sensors transform different forms of energy into readable electrical signals proportional to the physical quantity being measured [1]. Single-transduction-mechanism technology applies this fundamental concept for one-to-one conversion of signals in various sensing applications including in the biomedical field, environmental science, and energy harvesting [2,3,4,5]. Single transduction mechanism based sensors exhibit challenges such as limited ranges of detection and low signal-to-noise ratios [3]. These limitations arise due to reliance on a single parameter for information acquisition, despite the presence of more information available for quantification. The utilization of sensor array technology is a commonly employed approach to address these limitations of single-transduction systems [3,6,7].

Multi-transduction-mechanism technology is another approach used to address the existing limitations by combining more than one transduction mechanism into a single structure. This technology enables sensors to simultaneously convert the same or different forms of energy into different measurable electrical properties such as resistance and voltage [8,9]. This allows for broadened ranges of detection where the individual mechanisms operate in a range that supplements the other mechanisms to simultaneously distinguish between different conditions [10,11] As a result, multi-transduction technology can enhance a sensor’s signal-to-noise ratio, sensitivity, and selectivity beyond the performance of single-mechanism technology by incorporating multiple working principles within a single structure [10,11,12]. Moreover, this technology has the potential to reduce the number of required sensors in arrays, achieving miniaturization and further improving performance [13].

This paper introduces candidate devices that employ multi-transduction-mechanism technology by incorporating a combination of fundamental mechanisms into a single structure, rather than individual single-mechanism sensor arrays. The applications and advanced performance of multi-transduction sensors are emphasized by evaluating different possible transduction-mechanism combinations.

## 2. Fundamental Transduction Sensing Mechanisms

Fundamental transduction mechanisms are essential for detecting and responding to different types of stimuli. These mechanisms involve the conversion of energy into readable electrical signals. The common transduction mechanisms used in sensors include piezoelectricity, piezoresistivity, capacitive sensing, electromagnetic induction, and triboelectricity. The combination of these fundamental single mechanisms has the potential to create multi-transduction technology.

### 2.1. Piezoelectric Transduction

The piezoelectric effect is the reversable ability of certain dielectric materials to physically deform due to electric charges or generate electric charges in response to mechanical deformation [9]. When utilized for sensing, piezoelectric materials apply the latter property of this effect to convert mechanical energy, i.e., pressure, into electrical signals. Commonly used piezoelectric materials in sensors are lead zirconate titanate (PZT), barium titanate (BaTiO_3_), zinc oxide (ZnO) and polyvinylidene difluoride (PVDF), and aluminum nitride (AlN) [9,14]. The electrical signal output of the piezoelectric sensor is given by Equation (1) [15], where V is the voltage of the electrical signal output of the sensor, $d_{33}$ is the converse piezoelectric coefficient, C is the capacitance, and F is the applied pressure or mechanical change.

(1) $$V=\frac{d_{33}F}{C}$$

Piezoelectricity-based mechanisms offer high sensitivity on the order of pC/N [2,16], fast response time, mechanical flexibility, and chemical stability [3,4,14]. They are also active transducers and are thus self-powered [3,4]. However, these transducers are susceptible to high frequency noise due to their low signal-to-noise ratio, baseline drift, and temperature interference due to the pyroelectricity of piezo materials [3,4,14].

### 2.2. Piezoresistive Transduction

The piezoresistive effect is the change in resistance due to applied or external mechanical stimuli [8]. Silicon (Si) is the most commonly used material in piezoresistive devices. However, other materials such as germanium (Ge), silicon carbide (SiC), diamond, carbon nanotubes, and silicon nanowires have recently been explored to enhance properties such as temperature performance and sensitivity [17]. Piezoresistivity involves a change in resistance through two different mechanisms, namely, changes in geometrical parameters and electrical conductor resistance given by Equation (2) [8], or changes in material resistivity as in crystal semiconductors given by Equation (3) [8].

(2) $$R=\frac{k}{F}$$

(3) $$R=\frac{\rho l}{A}$$

In Equations (2) and (3), R, k, F, ρ, l, A represent electrical resistance, force, a constant having the dimension of Ω⋅kg, material resistivity, length of the conductor, and area, respectively.

Piezoresistive transducers are simple in design and have a wide range of detection-measuring pressures from Pa to kPa [18,19]. However, piezoresistive transducers are sensitive to temperature, and have high hysteresis and long relaxation times [3,4].

### 2.3. Capacitive Transduction

Capacitive transducers measure physical quantities by means of change in capacitance [20]. The simplest capacitive structure consists of two parallel plate electrodes separated by a dielectric material that can vary in separation, electrode area overlap, or electric permittivity [20]. Capacitive pressure sensors typically employ polydimethylsiloxane (PDMS) as the dielectric for their flexible and biocompatible properties [5,20,21], but can use other materials such as elastomers or silicon dioxide (SiO_2_) [22]. For soft electronics, electrode materials are commonly made of carbon nanotubes, graphene, carbon powders, gold, and metal nanowires [22,23,24]. The governing equation for capacitance-based transducers is given in Equation (4), where C is the capacitance of the transducer, A is the overlapping area of the two electrodes, d is the separation distance between the electrodes, $\varepsilon_{o}$ is the space permittivity, and $\varepsilon_{r}$ is the relative permittivity of the dielectric material [4].

(4) $$C=\frac{\varepsilon_{o}\varepsilon_{r}A}{d}\;$$

The advantages of capacitive transduction are in its simple governing equation, design and fabrication, and analysis [3,4]. However, these transducers are prone to hysteresis, and have limited miniaturization and relatively decreased sensitivity compared to piezoelectric and piezoresistive sensors [3,4,5,25].

### 2.4. Electromagnetic Induction Transduction

Electromagnetic induction is the measure of the change in the magnetic flux detected by a sensor coil given either the physical deformation of magnetic material [4,26], or the displacement of conductive material within a magnetic field [27], depending on the structure of the transducer [28]. Thin film magnetoelectric (ME) composites are used in micro-electromechanical system (MEMS) devices, tunable inductors, and magnetic sensors [29,30,31]. The composites consist of magnetostrictive and piezoelectric materials [29,31,32] such as ZnO, AlN, Metglas, BaTiO_3_, PVDF, and PZT [29,31]. The sensing principle of induction works by Faraday’s law in which an electromotive force is induced by a change in the magnetic flux [28]. For a static magnetic field, the induced electromotive force is shown in Equation (5) [28], where Ψ is the induced electromotive force, B is the magnetic flux density, and S is the closed surface area of the induction coil.

(5) $$\Psi =B\frac{dS}{dt}$$

The electrical readout for inductive transducers is the induced voltage, calculated as shown in Equation (6) [4], where ε is the induced voltage, N is the number of windings in the coil, and Φ is the magnetic flux.

(6) $$\varepsilon =-N\frac{d\Phi }{dt}$$

Induction-based sensors offer good sensitivity with magnetic resolution on the scale of nT [29,30] and simple fabrication, and are capable of wireless transmission [26,33]. However, inductive sensing requires conductive or ferromagnetic objects for detection and is less sensitive to humans or passive objects [34].

### 2.5. Triboelectric Transduction

Triboelectricity is a transduction mechanism that produces electricity as a result of contact friction between two materials of different triboelectric polarities [35,36]. When pairs of triboelectric material come into contact, electrons flow from the positive surface with lower electron affinity to the negative surface with higher electron affinity, resulting in a triboelectric effect [35].

The principle of triboelectrification itself is a challenge to quantitatively observe and reliably predict [36,37,38,39]. The effect holds for all states of matter and it is thus difficult to define a unified model that covers the properties of such diverse materials [37]. Triboelectrification can be influenced by a variety of factors, including the type and composition of the materials, the humidity and temperature of the environment, and the speed and pressure of the contact between the materials [40]. As a result, it is difficult to obtain repeatable measurements of generated triboelectric charges in a given situation [40]. Given these challenges, triboelectric transducers are commonly coupled with other transduction mechanisms such as electrostatic induction and piezoelectricity. These combinations are explored in the following section.

Suitable materials for triboelectric transduction technology have low friction coefficients <0.4 to reduce wear and heat, and high surface charge densities in units of mC/m^2^ to increase the ratio of collected charges per voltage unit [41]. The triboelectric materials with these properties include polyvinyl chloride (PVC), polytetrafluoroethylene (PTFE), and fluorinated ethylene propylene (FEP) [42].

Triboelectric transducers have shown promising results for applications such as human–machine interfaces, wearable electronic devices, robotics, prosthetic systems, wound healing, tactile sensing and e-skin, and e-noses, with its most prominent use as nanogenerators [42,43,44,45,46,47]. Due to their novelty, the shortcomings of triboelectric transducers are yet to be fully realized, but include limited short-circuit output current, post-stress conditions, structural deformation, and material property changes [48]. Research in different materials and transduction designs that employ multi-transduction-mechanism technology is currently underway to mitigate these effects.

## 3. Multi-Transduction Sensing Mechanisms

The technology of multi-transduction mechanisms involves more than one transduction mechanism within a single sensor structure, thereby enabling the sensor to detect and simultaneously distinguish between different types of stimuli for greater degrees of freedom [10,11]. The utilization of multi-transduction-mechanism technology allows a multi-parameter response and individual operation in different ranges, leading to an expanded range of operating conditions and improved performance [49,50,51]. Moreover, this technology has the potential to achieve miniaturization by reducing the number of utilized sensors in an array [13].

### 3.1. Combined Piezoresistive–Piezoelectric Transduction

Medical practitioners widely use the stethoscope for the examination of circulatory and respiratory systems through auscultation of body sounds from the heart and lungs. In traditional stethoscopes, the manual acoustic examination with sound is less reliable than that using electronic stethoscopes due to high interference leading to inaccuracy, and since no sensors are used, the sound volume is fixed [52]. Electronic stethoscopes consist of high-quality sensors and transducers that boost diagnostic capability by electronic amplification, thereby enabling important body sounds to be heard and reducing the possibility of missing vital clinical indicators of a patient’s condition. Hence, there is a need for advanced technology to improve the diagnostic power for efficient acquisition of bio-sounds.

**Figure 1:**
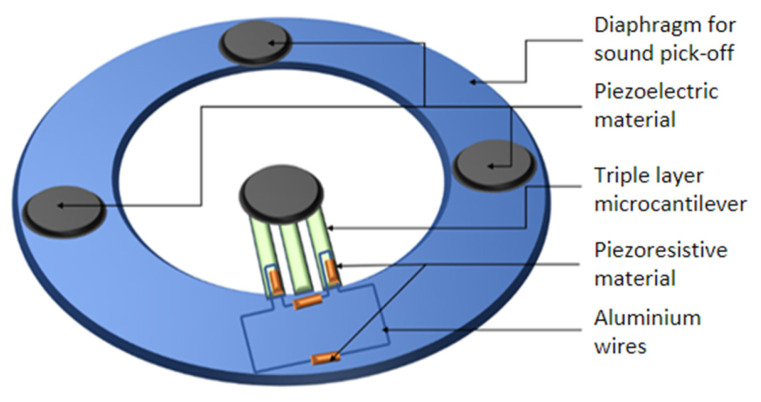
Schematic of a dual-transduction MEMS sensor employing piezoelectricity and piezoresistivity.

Conventional electronic stethoscopes are based on single-transduction-mechanism technology, which provides transmission of sound from the chest piece to the listener’s ears via air-filled tubing. A novel MEMS electronic stethoscope employing a dual-transduction mechanism is proposed by Pugazhenthi et al. [49]. The proposed dual-transduction device involves the use of piezoresistive and piezoelectric material to implement the pick-up of two frequency bands, which enables a broader bandwidth and high precision [49]. The proposed MEMS electronic stethoscope consists of a microcantilever [53] and microbeam, which are composed of magnets and piezoresistive and piezoelectric elements, as shown in Figure 1 [49].

The fixed part of the microcantilever consists of the piezoresistive elements and connecting aluminum wires. Piezoresistive transduction efficiency is dependent upon the materials chosen and the area of piezoresistive elements. Lightly doped silicon is utilized for piezoresistive elements in the reported design. The magnets are placed on the sound pick-up diaphragm. To generate magnetic inductive force, a magnet is placed at the free end of the cantilever. When sound waves are exerted on the diaphragm, magnets on the diaphragm deflect. The repulsive force is induced on the magnet placed on the cantilever, causing bending and periodic displacement of the cantilever, which results in a change in the resistance of the piezoresistors, as given by Equation (7) [53].

(7) $$\frac{\Delta R}{R}=\frac{\Delta \rho }{\rho }=\pi_{l}\sigma_{l}+\pi_{t}\sigma_{t}$$

ΔR, R, ρ, $\pi_{l}$ and $\pi_{t}$, $\sigma_{l}$, and $\sigma_{t}$, are the change in resistance, electrical resistance of the piezoresistive, electrical resistivity, longitudinal and transverse coefficients, and longitudinal and transverse stress, respectively.

The magnets placed on the top of the sound pick-up diaphragm have piezoelectric thin films fabricated on them. PZT-5H is the material utilized for the piezoelectric devices in the reported MEMS sensor design. Due to the deflection of the magnets when sound waves are exerted on the diaphragm and magnetic repulsive force, stress is developed on the top of piezoelectric elements. In MEMS piezoelectric devices, the relative directions of the electric field and strain constitute the mode of operation, i.e., $d_{31}$, where the electric field is perpendicular to the input strain/stress. The electric voltage developed across the two surfaces of the piezoelectric devices is given by Equation (8). The generated voltage V is proportional to the thickness t of the piezoelectric layer, the applied stress σ, and voltage constant $g_{31}$ of the piezoelectric material PZT-5H [54].

(8) $$V=tg_{31}\sigma \;$$

A finite element analysis was conducted to investigate the performance of the dual-transduction MEMS sensor in measuring heartbeat sound levels. The results indicated that the piezoresistive transduction produced sound levels ranging from 15 dB to 40 dB, while the piezoelectric transduction produced sound levels ranging from 40 dB to 90 dB. The reported signal-to-noise ratio (SNR) of the dual-transduction MEMS electronic stethoscope was found to be >17 dB for piezoelectric transduction and >15 dB for piezoresistive transduction, which represents an improvement over the conventional electronic stethoscope as reported in [55].

### 3.2. Combined Capacitive–Piezoresistive Transduction

**Figure 2:**
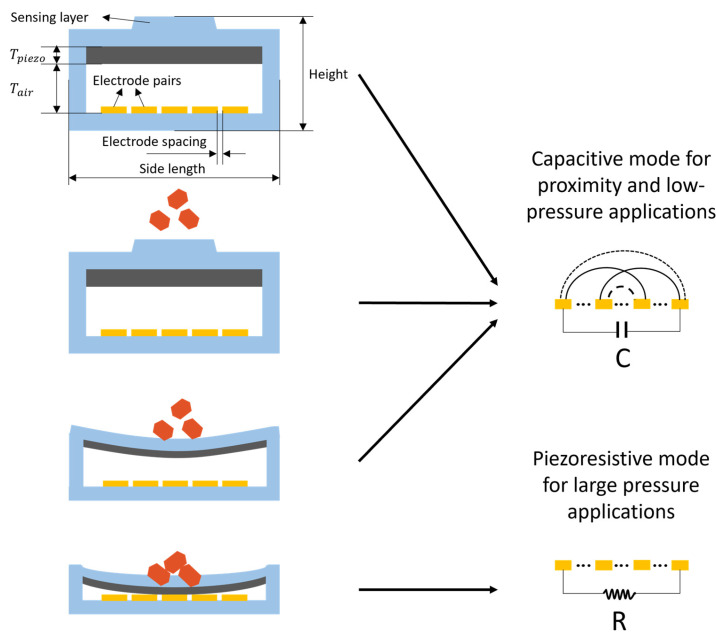
Working principle, design, and structural parameters of a capacitive–piezoresistive sensor for proximity and large pressure applications.

Electronic skins (e-skins) with multimodal perceptions have been developed to enable domestic and rehabilitation robots to interact with their environments more precisely, rapidly, and safely. These e-skins are mainly based on one or two mechanisms. A capacitive–piezoresistive hybrid sensor proposed by Ge et. al. was used for long-distance proximity and wide-range force detection in human–robot collaboration (HRC) and is a novel approach to improve e-skin performance [11]. Their hybrid sensor comprises of five layers: a silicone elastomer sealing layer with a bump in the center, a piezoresistive composite film formed by carbon black fillers in the PDMS matrix, two interdigital copper foil electrodes, an air gap between the piezoresistive film and the electrode, and a silicone elastomer substrate, as shown in Figure 2 [11]. Specifically, two interdigital electrodes are employed to form a coplanar capacitor with its upper space.

The side length, height of the sensor unit, number of electrode pairs, electrode spacing, thickness of the air gap, and piezoresistive film are the design parameters of the sensor. The capacitance $C_{o}$ and resistance $R_{o}$, as well as their relative changes $\frac{\Delta C}{C_{o}}$ and $\frac{\Delta R}{R_{o}}$, were taken as optimization targets for the sensitivity and selectivity of the sensor.

The novel sensor with capacitive and piezoresistive modes for proximity and contact force sensing has four operating conditions: the initial condition, approaching condition, initial contact condition, and considerable pressure condition. The novel sensor switches between the capacitive and piezoresistive modes for proximity and contact force sensing, depending on the condition, as shown in Figure 2 [11]. In the initial condition with no external object approaching, the electric field distribution between the coplanar electrode will not be changed. Since there is no deformation of the structure, the electrical capacitance and resistance remain unchanged. When an external object approaches, a gradual change in potential and distribution of the electric field above the coplanar electrode occurs, leading to a change in the sensor capacitance. In this scenario, the capacitive mode is the response for the proximity detection [11]. Once an object makes contact with the bump, the contact force will induce the deformation of the structures above the coplanar electrode, as shown in Figure 2. The electric field lines are mainly restrained within the PDMS cover and air gap above the electrode. The deformation, including the compression of piezoresistive film, PDMS cover, and air gap, will lead to change in the capacitance [11]. At this stage, the device still works in capacitive mode for these faint pressures. Further increasing the force results in greater deformation of the piezoresistive film, resulting in its contact with the coplanar electrode. In this scenario, the operating model has been changed to a resistive mode owing to the conduction of piezoresistive film between two electrodes [11]. Above all, the proposed sensor structure can detect the proximity of the external object as well as the contact force using hybrid capacitive–piezoresistive modes.

The change in the sensing mechanism from the capacitive mode to the piezoresistive mode extended the sensitivity range of applied force to 450 N [11]. In addition, the high stability for both proximity and contact force sensing guarantees practical usage in robots. Finally, the e-skins are used for safety control in HRC and exhibit capabilities of long-distance proximity and large-range force detection, illustrating their potential to meet rigorous safety requirements that demand complex and precise sensing solutions [11].

### 3.3. Combined Capacitive–Piezoelectric Transduction

The two prominent MEMS ultrasonic transducers, the capacitive micromachined ultrasonic transducer (CMUT) and the piezoelectric micromachined ultrasonic transducer (PMUT), are known for their applications in medical imaging, non-destructive testing, acoustic power transmissions, etc., wherein operation frequencies and choice of devices are application-dependent [9]. The commonly employed frequency range is 20 kHz–40 MHz [56]. Ultrasound transducers operating at higher frequencies (in the MHz range) suffer from low penetration depths, frequency-dependent attenuation, and low depth of field [56]. Using low-frequency transducers that operate in the kHz range can overcome this disadvantage but leads to issues of reduced resolution [50,56]. The idea of dual-frequency ultrasonic transducers capable of operating in both high and low frequencies has therefore been explored to overcome these issues by utilizing arrays of PMUTs [6] or CMUTs [7] with different dimensions fabricated on the same substrate. This mode of implementation introduces acoustic crosstalk between low- and high-frequency elements of the array [6,7].

The combination of the CMUT and PMUT operation modes in a dual-frequency ultrasonic transducer (DFUT) has been proposed by Sun et al. [50] to overcome this challenge. The three-layer device comprises of a piezoelectric layer placed atop the flexible membrane of a CMUT, enabling both high- and low-frequency modes of operation.

**Figure 3:**
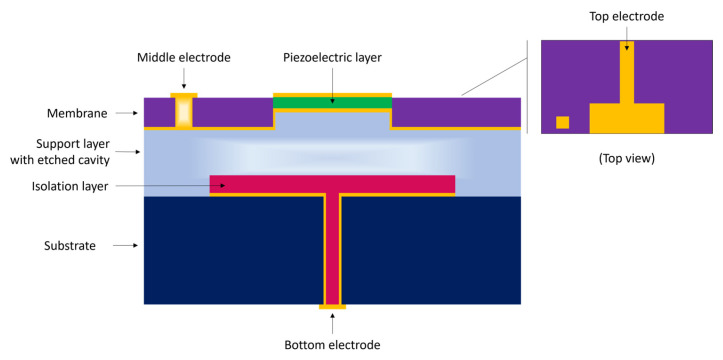
Schematic diagram of capacitive–piezoelectric DFUT.

The dual-frequency ultrasound transducer is composed of a large elastic layer with a small, etched cavity, a piezoelectric layer on top of the elastic layer, and three conductive metal layers serving as electrodes on the top, middle, and bottom, as shown in Figure 3 [50]. When a low-frequency AC voltage with a DC bias is applied between the middle and bottom electrodes, the device functions as a CMUT with an operating frequency of 5.9 MHz. When a high-frequency AC voltage is applied between the top and middle electrodes, the piezoelectric layer deforms and produces a 26.6 MHz high-frequency acoustic wave similar to a PMUT. To ensure stable and reliable dual-frequency operation, a small cavity is etched into the central laminated layers, leaving a thin membrane to support the piezoelectric layer and soften the mechanical stiffness of the layers. This allows the outer elastic layer to define the square-shaped clamped boundaries of the central layers when they vibrate at the PMUT mode.

The governing equation for the CMUT mode of operation is approximated by Equation (9) [50] considering only the large elastic plate is clamped and the external load term is neglected [6]. This provides the solution for the first bending moment of the DFUT in vacuum, where $D_{c}$ and $m_{c}$ are the equivalent flexural rigidity and the average mass per unit area of the whole laminated plates, respectively; l is the length of the large elastic plate; $\lambda_{c}$ is the vibration mode parameter of the laminated plates [6].

(9) $$f_{1,C}=\frac{\lambda_{c}}{2\pi l^{2}}\sqrt{\frac{D_{c}}{m_{c}}}$$

The first eigenfrequency of the DFUT operating in the high-frequency PMUT mode is calculated using a similar method assuming the outer large elastic layer is built-in and only the central piezoelectric laminated layers vibrate, as approximated in Equation (10) [50], where $D_{p}$ and $m_{p}$ are the equivalent flexural rigidity and the average mass per unit area of the piezoelectric laminated plates, respectively; a is the length of the piezoelectric plate.

(10) $$f_{1,P}=\frac{18}{\pi a^{2}}\sqrt{\frac{D_{p}}{m_{p}}}$$

Compared to conventional standalone CMUTs and PMUTs of equal dimensions, the hybrid DFUT yields a good dual-frequency operation due to the amplitude order matching of the high-frequency and low-frequency components [50]. The DFUT can operate at both low frequency (5.9 MHz) and high frequency (26.6 MHz), enabling it to provide a high resolution of <200 µm and wide detection distance of >3 cm through its multi-transduction-mechanism technology [50]. In contrast, a standalone PMUT can provide high-resolution imaging but has limited detection distance, while a standalone CMUT can offer a wider detection distance but lower resolution. Therefore, the DFUT’s ability to combine the benefits of both CMUT and PMUT modes of operation suggests the potential for the implementation of single-element ultrasound transducers capable of producing both high lateral resolution and larger imaging depths [50].

### 3.4. Hybrid Nanogenerators

Advancements in miniaturized integrated sensing platforms have seen demand for their use in wearable devices, biomedical implants, and wireless sensor networks. One of the major challenges faced in such applications is the energy source. Chemical batteries such as lithium ion (Li-ion) are not ideal due to drawbacks such as their fixed energy density, short lifespan, and bulky nature [57]. Moreover, they pose additional risks such as the release of heat during thermal events, toxic exposure in the case of electrolyte leakage, and device malfunction due to battery depletion [57]. A viable alternative while mitigating these risks can be found in the form of energy harvesters.

Nanogenerators have been designed by employing a single-transduction mechanism, commonly the piezoelectric effect, the electrostatic effect, electromagnetic induction, and more recently, the triboelectric effect [58,59,60]. Traditional vibrational energy harvesters based on single transduction mechanisms can provide output voltages of a few hundred microvolts and are only capable of producing a few microwatts of power [61]. To improve the performance metrics of stand-alone transducers, hybrid energy harvesters based on multi-transduction mechanisms have been increasingly researched in recent years. The commonly combined mechanisms used in hybrid energy harvesters include triboelectric–inductive, piezoelectric–triboelectric, and piezoelectric–inductive.

#### 3.4.1. Combined Triboelectric–Inductive Transduction

The most prominent use of triboelectric transduction is the triboelectric nanogenerator (TENG). First proposed by Wang and his team in 2012, TENG takes advantage of the charge generation due to the coupling of the triboelectrification effect and electrostatic induction. By separating materials via applied periodic mechanical force, current is induced through an external circuit [35]. The governing equation of the TENG demonstrates the relationship between voltage, charge, and separation distance (V-Q-X), as shown in Equation (11) [47,62,63].

(11) $$V=-\frac{1}{C\left(X\right)}Q+V_{OC}\left(X\right)$$

V represents the total voltage from the inherent capacitance between electrodes and the open-circuit voltage, $V_{OC}\left(X\right)$, resulting from polarized triboelectric charges, X is the distance between triboelectric layers, $C\left(X\right)$ is the capacitance between the electrodes, and Q is the output charge transferred from one electrode to the other [4,62,63].

**Figure 4:**
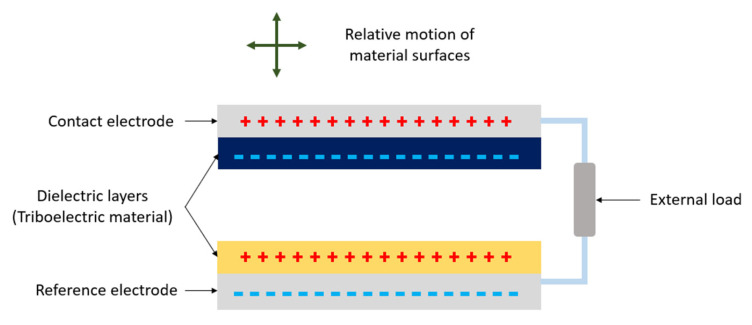
Schematic representation of TENG working modes. The relative motion between the triboelectric materials determines the mode of operation.

TENG has since developed into four fundamental operation modes, each with their own application circumstances. The general structure of TENGs shared between the modes, as shown in Figure 4 [42,47], has two electrodes separated by two dielectrics for contact electrification and connected to an external load [47].

Contact-separation mode alternates between the connection and separation of materials with distinct electron affinities to induce alternating current and is the most common mode of operation for TENGs [44]. The mode is a simple structure with low wear that is easy to fabricate but difficult to package due to its varying volume gap [44]. In linear-sliding mode, the relative sliding of the triboelectric material pair induces polarization, which can work in higher frequencies but can reduce durability with prolonged usage [44,64]. On the other hand, single-electrode mode configures a single stationary electrode that serves as the triboelectric layer and employs a dielectric component that is free to move towards or away from the electrode to transfer charges [44,64]. The dielectric serves as an electric potential reference that is not bounded to the electrode, thus simplifying its fabrication and improving operation convenience, but lowering output performance [44,65]. Freestanding triboelectric layer mode contains a pair of symmetric electrodes connected to each other with a load in a stationary, ground-free configuration that allows external objects to freely move between them to generate potential [44,64].

The common property shared amongst triboelectric transducers, regardless of their characteristic working principles, is that the devices are self-powered. They can harvest irregular, low-frequency energy from mechanical or chemical stimuli and convert it into electrical energy by contact and relative motion in the range of 1~100 Hz [42,60], through means of coupled triboelectrification and electrostatic induction [37,42]. This quality, along with their low cost, portability, material robustness, and customizable structure, creates the potential for improved active sensors, energy harvesters, driving actuators, and microsystems to meet the demands for power-efficient, miniaturized, and discrete electronic devices [35,44,64,66]. However, practical applications of TENGs are limited due to their high output voltages (~1–1000 V) and low current (~1–1000 µA) [60], and they are thus further combined with other transduction mechanisms such as piezoelectricity.

#### 3.4.2. Combined Piezoelectric–Triboelectric Transduction

Piezoelectric and triboelectric nanogenerators have become popular approaches applied to wearable sensors [67], but have since been combined to create hybrid piezo-triboelectric nanogenerators [58]. Epidermal piezo-triboelectric hybrid nanogenerators have been applied to cardiovascular, respiratory, and neurological monitoring [10], as well as motion tracking for real-time posture correction [68], human–machine interfaces, and robotics [51] to effectively harvest the mechanical energy of human motion. Liu et al. demonstrated how these devices can be used to detect faint body movements from the micro-amperage current resulting from the bending and stretching motions when placed on one’s arm or the back of the hand [68]. The ability to detect low-frequency mechanical vibrations can be attributed to the narrow gap between triboelectric pairs and the high sensitivity of the film [68]. When a force is applied to the device, the separated PDMS and silk nanofibers come into contact and put pressure on each other, operating in a similar method to that of triboelectric contact separation mode [68]. Electrons flow towards the PDMS from the more triboelectric positive silk, creating a potential difference between the pairs and inducing current [68]. An additional current pulse is generated at the same time due to the piezoelectricity of the material [68]. When the force is removed, all components return to their original state, generating current in the opposite direction [68]. Varying the frequency of contract separation resulted in different output performance, where higher frequency resulted in faster energy harvest and a greater striking force [68].

The hybrid piezo-triboelectric wearable sensor proposed by Mariello et al. takes advantage of the complementary effects of piezoelectricity, skin-contact actuation, and piezo-triboelectric hybrid contact [51]. The researchers claim stable and repeatable bio-signal detection with consideration of sudden motions and micro-friction due to skin deformations [51]. The prototype, made of three inorganic and elastomeric electrodes, harnesses contact electrification between the piezo-triboelectric sensor, as well as between the triboelectric sensor and the user’s skin. This configuration was assembled using double-sided adhesive tape to set the piezo- and tribosensors facing each other [51]. Individual signals from each sensor were detected simultaneously and passed through two series-connected, full-bridge Schottky-diode rectifiers to measure their hybrid output signal [51]. In application, the user’s skin comes into contact with the device through an ultra-soft patch covered on both sides by a thin, friction parylene film [51]. Mariello et al. evaluated their device for gait walking, hand and finger gestures, and joint movements, and observed a wider range of measurements for faint and irregular motions. The combination of piezoelectricity and triboelectricity mechanisms allowed detection at low- and medium-pressure ranges of 0–50 kPa and 50–120 kPa [51]. In the low-pressure range, the triboelectric effect dominates and results in a sensitivity of 59.4 mV kPa^−1^ due to the high deformability of the triboelectric sensor compared to the piezoelectric sensor, whereas in the medium-pressure ranges, when both mechanisms work together, the sensitivity increases to 160 mV kPa^−1^ [51].

**Figure 5:**
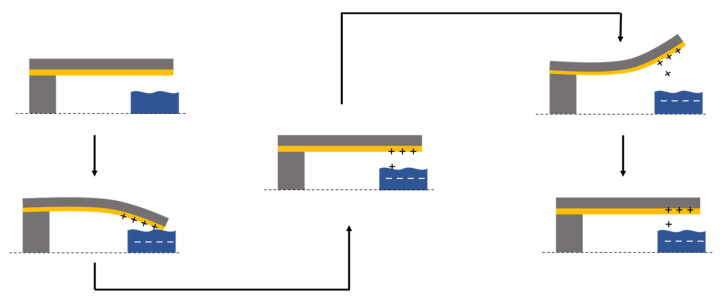
Working principle of a hybrid piezoelectric triboelectric nanogenerator based on a cantilever structure.

A low-frequency wide-band hybrid energy harvester proposed by Han et al. [69] combines a piezoelectric energy harvester and a TENG together to effectively harvest low-frequency vibrational energy, as shown in Figure 5 [69]. A polyvinylidene fluoride (PVDF) cantilever array consisting of 3 cantilevers of varying lengths (25, 16, and 12 mm) coupled with a TENG consisting of a micro/nano dual-scale (MNDS) polydimethylsiloxane (PDMS) is utilized to produce the piezoelectric and triboelectric outputs, respectively. When a vibration excitation is induced, the cantilever undergoes deflection towards the MNDS PDMS, and the Al bottom electrode of the cantilever collides with the MNDS PDMS surface. The bending of the cantilever produces a $d_{31}$ piezoelectric output, while the collision electron transfer between the surfaces takes place. The recovery of the cantilever to its original state generates a triboelectric output through a connected external load due to the increase in the gap distance between the cantilever and the MNDS PDMS. The deflection in the opposite direction generates a reversed piezoelectric output and a triboelectric output due to the continued increase in the distance between the two surfaces. The cantilever then recovers to its original state, closing the gap between the two surfaces, thereby producing a reversed triboelectric output.

#### 3.4.3. Combined Piezoelectric–Inductive Transduction

**Figure 6:**
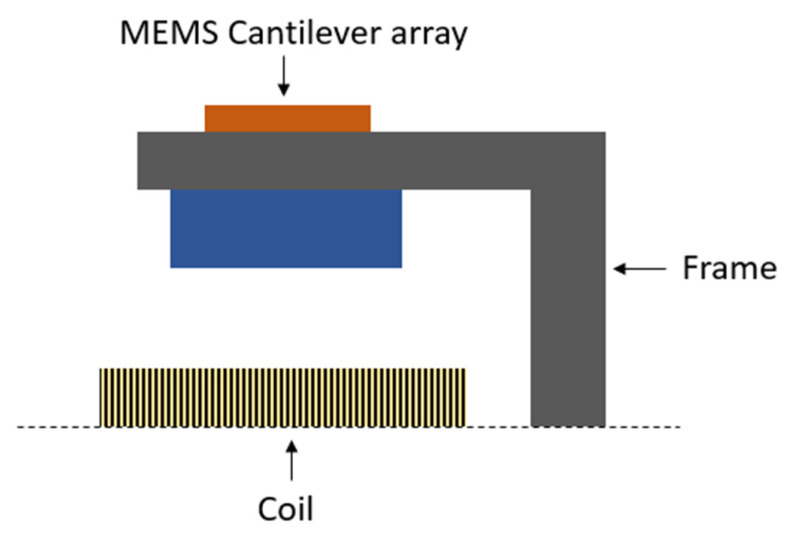
Schematic diagram of hybrid energy harvester.

A hybrid vibrational energy harvester, presented by Yu et al., is based on a combination of piezoelectric and electromagnetic modes of transduction [61]. The device consists of a MEMS PZT cantilever array, a neodymium (NdFeB) permanent magnet attached to the free end of the cantilever array, and one multilayer printed circuit board (PCB) coil placed on the base, as depicted in Figure 6 [61].

A mathematical model for the total energy converted from mechanical vibrations to electrical energy was provided, which is essentially a second-order, spring-mass-damper system and is given by Equation (12) [61].

(12) $$P_{hybrid}=P_{Piezo}+P_{EM}=\frac{9}{64}\frac{E_{ac}}{L^{2}\zeta {\;}_{T}\omega^{2}}+\frac{{\left(NlB\omega Y\right)}^{2}}{16\zeta {\;}_{T}^{2}R_{coil}}$$

$\zeta {\;}_{T}$ is the overall damping factor of the system, $E_{ac}$ is the modulus of the piezoelectric unimorph cantilever beam, ω is the resonant frequency of the system, Y is the amplitude of the vibration source, N is the number of turns of the coil, L is the length of coil, and B is the magnetic flux density. Based on this model, it can be determined that the output of the combined system would be higher than that of the individual transduction mechanisms, but lower than the sum of their outputs due to an increase in total damping of the device.

The device was tested with a vibration exciter and the output was measured using an oscilloscope. Comparison of the results from the hybrid transducer with its stand-alone piezoelectric and electromagnetic counterparts for an excitation acceleration of 0.2 g revealed a total maximum power of 40.62 µW, which was 13% and 147% higher than the outputs from the stand-alone piezoelectric (35.82 µW) and electromagnetic (16.44 µW) energy harvesting arrays, respectively. True to the model, the sum of the individual device outputs is greater than the output of the hybrid device. Utilizing this combined mode of transduction, this device also showed a lower operational frequency due to an increase in the overall structural damping factor of the device. The published resonant frequency of the hybrid energy harvester is 55.9 Hz, which is less than that of the individual devices at 56.3 Hz and 56.2 Hz for the piezoelectric and electromagnetic energy harvesters, respectively [61].

Variants of the above design are the most widespread hybrid energy harvesters for the combination of piezo and electromagnetic transduction wherein parameters such as the orientation of the magnet with respect to the coil, suspension of the magnet on the PZT, structure of the piezoelectric beam, and dimensions of the components are varied [70,71,72,73,74,75,76]. The above results provide insight into the potential to draw improved energy harvesting performance through the combination of piezoelectric and electromagnetic modes of transduction.

### 3.5. Summary of Combined Transduction Mechanisms

The possible transduction mechanism combinations include triboelectric–inductive, piezoelectric–triboelectric, piezoelectric–inductive, piezoelectric–capacitive, and capacitive–piezoresistive. The impact of these combined transduction mechanisms on performance is governed by key parameters, which are summarized in Table 1.

A dual-frequency ultrasonic transducer (DFUT) that merges a CMUT and PMUT to produce high lateral resolution and larger imaging depth was proposed [50]. Another study reported combined capacitive–piezoresistive and combined piezoelectric–triboelectric transduction mechanisms, which showed potential for increasing the range of detection by expanding the operational range [11,51]. Furthermore, the combined piezoresistive–piezoelectric transduction enables a broader frequency bandwidth resulting in improved signal-to-noise ratio [49].

The combination of transduction mechanisms, including combined triboelectric–inductive transduction, shows the ability to harvest low-frequency energy [42,60]. Additionally, the reported combined piezoelectric–inductive transduction shows potential for harvesting energy by increasing the total maximum energy converted from mechanical vibrations to electrical energy [61].

## 4. Conclusions

This paper presents the working principles, governing equations, advantages, and challenges of the fundamental transduction mechanism candidates used in the development of multi-transduction-mechanism technology. These mechanisms include piezoelectric, piezoresistive, capacitive, inductive, and triboelectric transduction mechanisms. The method commonly used to address the challenges of single-transduction systems is the utilization of sensor arrays [3,6,7]. These challenges include limited ranges of detection and low signal-to-noise ratios [3].

Multi-transduction-mechanism technology is a novel methodology used to enhance performance and reduce the number of sensors within an array. The possible transduction mechanism combinations include triboelectric–inductive, piezoelectric–triboelectric, piezoelectric–inductive, piezoelectric–capacitive, and capacitive–piezoresistive. The technology of the multi-transduction mechanism offers improved performance compared to single transduction mechanism based devices by incorporating multiple working principles into a single structure. The resulting increased degrees of freedom provide additional information for a broader range of operating conditions. This is achieved by simultaneously measuring multi-parameter electrical outputs from the conversion of energy sources under the same environmental conditions, and via individual transduction mechanism operation in different ranges [49,50,51]. Furthermore, this technology shows its potential to achieve miniaturization by reducing the number of required sensors [13].

Through further development, the use of multi-transduction mechanisms in the next generation of sensors is expected to be prevalent, particularly in applications such as imaging, sensing, and energy harvesting. 

## Figures and Tables

**Table 1:** The summary of combined transduction mechanisms.

Combined Transduction Mechanisms	Key Parameters	Effect on Performance	Reference (s)
Piezoresistive–Piezoelectric	Resistance R, Voltage V	Improved signal-to-noise ratio	[49]
Capacitive–Piezoresistive Transduction	Capacitance C, Resistance R	Long-distance proximity, large-range force detection	[11]
Capacitive–Piezoelectric (DFUT)	CMUT frequency $f_{C}$*,* PMUT frequency $f_{P}$	High resolution (>200 μm), larger imaging depths (>3 cm)	[50]
Triboelectric–Inductive	Charge Q*,* Separation distance between triboelectric layers X	Harvest low-frequency energy in the range of [1~100 Hz]	[44,65]
Piezoelectric–Triboelectric	Voltage V*,* Charge Q	Detection at low [0~50 kPa] and medium [50~120 kPa] pressure ranges, Increased sensitivity to $160\;{\mathrm{mV}\;\mathrm{kPa}}^{-1}$ in the medium pressure ranges.	[51]
Piezoelectric–Inductive	Piezoelectric power $P_{Piezo}$*,* Electromagnetic power $P_{EM}$	Increased power to 40.62 µW for energy harvesting	[61]

## Data Availability

No new data were created or analyzed in this study. Data sharing is not applicable to this article.

## References

[1] Sinclair I.R. (2001). Sensors and Transducers.

[2] Gong S., Zhang B., Zhang J., Lin Wang Z., Ren K., Gong S., Zhang B., Zhang J., Wang Z.L., Ren K. (2020). Biocompatible Poly(Lactic Acid)-Based Hybrid Piezoelectric and Electret Nanogenerator for Electronic Skin Applications. Adv. Funct. Mater..

[3] dos Santos A., Fortunato E., Martins R., Águas H., Igreja R. (2020). Transduction Mechanisms, Micro-Structuring Techniques, and Applications of Electronic Skin Pressure Sensors: A Review of Recent Advances. Sensors.

[4] Meng K., Xiao X., Wei W., Chen G., Nashalian A., Shen S., Xiao X., Chen J. (2022). Wearable Pressure Sensors for Pulse Wave Monitoring. Adv. Mater..

[5] Kumaresan Y., Ozioko O., Dahiya R. Effect of Dielectric and Stiffness of Soft Material between the Electrodes of a Capacitive Pressure Sensor on Its Performance. Proceedings of the FLEPS 2020—IEEE International Conference on Flexible and Printable Sensors and Systems.

[6] Liu L., Ji W., Xing Z., Sun X., Chen Y., Du Y., Qin F. (2021). A Dual-Frequency Piezoelectric Micromachined Ultrasound Transducer Array with Low Inter-Element Coupling Effects. J. Micromechanics Microengineering.

[7] Maadi M., Ceroici C., Zemp R.J. (2021). Dual-Frequency CMUT Arrays for Multiband Ultrasound Imaging Applications. IEEE Trans. Ultrason. Ferroelectr. Freq. Control..

[8] Fiorillo A.S., Critello C.D., Pullano A.S. (2018). Theory, Technology and Applications of Piezoresistive Sensors: A Review. Sens. Actuators A Phys..

[9] Birjis Y., Swaminathan S., Nazemi H., Raj G.C.A., Munirathinam P., Abu-Libdeh A., Emadi A. (2022). Piezoelectric Micromachined Ultrasonic Transducers (PMUTs): Performance Metrics, Advancements, and Applications. Sensors.

[10] Yang Y., Wang Z.L., Mariello M. (2022). Recent Advances on Hybrid Piezo-Triboelectric Bio-Nanogenerators: Materials, Architectures and Circuitry. Nanoenergy Adv..

[11] Ge C., Wang Z., Liu Z., Wu T., Wang S., Ren X., Chen D., Zhao J., Hu P., Zhang J. (2022). A Capacitive and Piezoresistive Hybrid Sensor for Long-Distance Proximity and Wide-Range Force Detection in Human–Robot Collaboration. Adv. Intell. Syst..

[12] Zhang J., He Y., Boyer C., Kalantar-Zadeh K., Peng S., Chu D., Wang C.H. (2021). Recent Developments of Hybrid Piezo–Triboelectric Nanogenerators for Flexible Sensors and Energy Harvesters. Nanoscale Adv..

[13] Sberveglieri G., Genzardi D., Greco G., Nunez-Carmona E., Pezzottini S., Sberveglieri V. (2022). The Electronic Nose: Review on Sensor Arrays and Future Perspectives. Chem. Eng. Trans..

[14] Park J., Kim M., Lee Y., Lee H.S., Ko H. (2015). Nanomaterials: Fingertip Skin-Inspired Microstructured Ferroelectric Skins Discriminate Static/Dynamic Pressure and Temperature Stimuli. Sci. Adv..

[15] Rovisco A., Dos Santos A., Cramer T., Martins J., Branquinho R., Águas H., Fraboni B., Fortunato E., Martins R., Igreja R. (2020). Piezoelectricity Enhancement of Nanogenerators Based on PDMS and ZnSnO3Nanowires through Microstructuration. ACS Appl. Mater. Interfaces.

[16] Zhang Z.H., Kan J.W., Yu X.C., Wang S.Y., Ma J.J., Cao Z.X. (2016). Sensitivity Enhancement of Piezoelectric Force Sensors by Using Multiple Piezoelectric Effects. AIP Adv..

[17] Winter P.M., Lanza G.M., Wickline S.A., Madou M., Wang C., Deotare P.B., Loncar M., Yap Y.K., Rose J., Auffan M. (2012). Piezoresistivity. Encyclopedia of Nanotechnology.

[18] Wang M., Zhang H., Wu H., Ma S., Ren L., Liang Y., Liu C., Han Z. (2022). Bioinspired Flexible Piezoresistive Sensor for High-Sensitivity Detection of Broad Pressure Range. Bio-Des. Manuf..

[19] Li J., Wu T., Jiang H., Chen Y., Yang Q. (2021). Ultrasensitive Hierarchical Piezoresistive Pressure Sensor for Wide-Range Pressure Detection. Adv. Intell. Syst..

[20] Rivadeneyra A., López-Villanueva J.A. (2020). Recent Advances in Printed Capacitive Sensors. Micromachines.

[21] Masihi S., Panahi M., Maddipatla D., Hanson A.J., Bose A.K., Hajian S., Palaniappan V., Narakathu B.B., Bazuin B.J., Atashbar M.Z. (2021). Highly Sensitive Porous PDMS-Based Capacitive Pressure Sensors Fabricated on Fabric Platform for Wearable Applications. ACS Sens..

[22] Ullah H., Wahab M.A., Will G., Karim M.R., Pan T., Gao M., Lai D., Lin Y., Miraz M.H. (2022). Recent Advances in Stretchable and Wearable Capacitive Electrophysiological Sensors for Long-Term Health Monitoring. Biosensors.

[23] McCoul D., Hu W., Gao M., Mehta V., Pei Q., McCoul D., Hu W., Gao M., Mehta V., Pei Q. (2016). Recent Advances in Stretchable and Transparent Electronic Materials. Adv. Electron. Mater..

[24] Pagoli A., Chapelle F., Corrales-Ramon J.A., Mezouar Y., Lapusta Y. (2022). Large-Area and Low-Cost Force/Tactile Capacitive Sensor for Soft Robotic Applications. Sensors.

[25] Kim S.W., Oh G.Y., Lee K.I., Yang Y.J., Ko J.B., Kim Y.W., Hong Y.S. (2022). A Highly Sensitive and Flexible Capacitive Pressure Sensor Based on Alignment Airgap Dielectric. Sensors.

[26] Chang H.C., Liao S.C., Cheng C.L., Wen J.H., Hsieh H.S., Lai C.H., Fang W. Wireless Magnetostrictive Type Inductive Sensing CMOS-MEMS Pressure Sensors. Proceedings of the IEEE International Conference on Micro Electro Mechanical Systems (MEMS).

[27] Kinsler P. (2020). Faraday’s Law and Magnetic Induction: Cause and Effect, Experiment and Theory. Physics.

[28] Liu S., Xu H., Xu D., Xiong B. (2017). Modelling of Resonant MEMS Magnetic Field Sensor with Electromagnetic Induction Sensing. Solid. State Electron..

[29] Liang X., Chen H., Sun N.X. (2021). Magnetoelectric Materials and Devices. APL Mater..

[30] Gao J., Jiang Z., Zhang S., Mao Z., Shen Y., Chu Z. (2021). Review of Magnetoelectric Sensors. Actuators.

[31] Mao Q., Wu J., Hu Z., Xu Y., Du Y., Hao Y., Guan M., Wang C., Wang Z., Zhou Z. (2021). Magnetoelectric Devices Based on Magnetoelectric Bulk Composites. J. Mater. Chem. C. Mater..

[32] Bichurin M., Sokolov O., Ivanov S., Leontiev V., Petrov D., Semenov G., Lobekin V. (2022). Physics of Composites for Low-Frequency Magnetoelectric Devices. Sensors.

[33] Byberi A., Amineh R.K., Ravan M. (2022). Wearable Inductive Sensing of the Arm Joint: Comparison of Three Sensing Configurations. Magnetism.

[34] Moheimani R., Hosseini P., Mohammadi S., Dalir H. (2022). Recent Advances on Capacitive Proximity Sensors: From Design and Materials to Creative Applications. C.

[35] Zhao Z., Lu Y., Mi Y., Meng J., Cao X., Wang N. (2022). Structural Flexibility in Triboelectric Nanogenerators: A Review on the Adaptive Design for Self-Powered Systems. Micromachines.

[36] Triboelectricity – MRSEC Education Group – UW–Madison. (n.d.). https://education.mrsec.wisc.edu/triboelectricity/.

[37] Wang Z.L., Wang A.C. (2019). On the Origin of Contact-Electrification. Mater. Today.

[38] Can Triboelectric Nanogenerators Find Their Niche? (n.d.). https://cen.acs.org/materials/energy-storage/triboelectric-nanogenerators-find-niche/99/i18.

[39] Zhang R., Olin H. (2020). Material Choices for Triboelectric Nanogenerators: A Critical Review. Ecomat.

[40] Zou H., Zhang Y., Guo L., Wang P., He X., Dai G., Zheng H., Chen C., Wang A.C., Xu C. (2019). Quantifying the Triboelectric Series. Nat. Commun..

[41] Zhao Z., Zhou L., Li S., Liu D., Li Y., Gao Y., Liu Y., Dai Y., Wang J., Wang Z.L. (2021). Selection Rules of Triboelectric Materials for Direct-Current Triboelectric Nanogenerator. Nat. Commun..

[42] Kim D.W., Lee J.H., Kim J.K., Jeong U. (2020). Material Aspects of Triboelectric Energy Generation and Sensors. NPG Asia Mater..

[43] Song Z., Yin J., Wang Z., Lu C., Yang Z., Zhao Z., Lin Z., Wang J., Wu C., Cheng J. (2022). A Flexible Triboelectric Tactile Sensor for Simultaneous Material and Texture Recognition. Nano Energy.

[44] Pu X., An S., Tang Q., Guo H., Hu C. (2021). Wearable Triboelectric Sensors for Biomedical Monitoring and Human-Machine Interface. iScience.

[45] Chen X., Xie X., Liu Y., Zhao C., Wen M., Wen Z. (2020). Advances in Healthcare Electronics Enabled by Triboelectric Nanogenerators. Adv. Funct. Mater..

[46] Ha M., Park J., Lee Y., Ko H. (2022). Triboelectric Generators and Sensors for Self-Powered Wearable Electronics. ACS Nano.

[47] Wu C., Wang A.C., Ding W., Guo H., Wang Z.L. (2019). Triboelectric Nanogenerator: A Foundation of the Energy for the New Era. Adv. Energy Mater..

[48] Godwinraj D., George S.C. (2021). Recent Advancement in TENG Polymer Structures and Energy Efficient Charge Control Circuits. Adv. Ind. Eng. Polym. Res..

[49] Sundararajan A.D.D., Pugazhenthi R. (2022). Design of Dual Transduction-Based CMOS-MEMS Electronic Stethoscope. ECS Trans..

[50] Sun C., Dai F., Jiang S., Liu Y. A Novel Single-Element Dual-Frequency Ultrasound Transducer for Image-Guided Precision Medicine. Proceedings of the IEEE International Ultrasonics Symposium, IUS.

[51] Mariello M., Fachechi L., Guido F., De Vittorio M., Mariello M., Fachechi L., Guido F., De Vittorio M. (2021). Conformal, Ultra-Thin Skin-Contact-Actuated Hybrid Piezo/Triboelectric Wearable Sensor Based on AlN and Parylene-Encapsulated Elastomeric Blend. Adv. Funct. Mater..

[52] Kalinauskienė E., Razvadauskas H., Morse D.J., Maxey G.E., Naudžiūnas A. (2019). A Comparison of Electronic and Traditional Stethoscopes in the Heart Auscultation of Obese Patients. Medicina (Kaunas).

[53] Park S.J., Doll J.C., Pruitt B.L. (2010). Piezoresistive Cantilever Performance—Part I: Analytical Model for Sensitivity. J. Microelectromech Syst..

[54] Kim S.G., Priya S., Kanno I. (2012). Piezoelectric MEMS for Energy Harvesting. MRS Bull..

[55] Rennoll V., McLane I., Emmanouilidou D., West J., Elhilali M. (2021). Electronic Stethoscope Filtering Mimics the Perceived Sound Characteristics of Acoustic Stethoscope. IEEE J. Biomed. Health Inform..

[56] Zang J., Fan Z., Li P., Duan X., Wu C., Cui D., Xue C. (2022). Design and Fabrication of High-Frequency Piezoelectric Micromachined Ultrasonic Transducer Based on an AlN Thin Film. Micromachines.

[57] Taalla R.V., Arefin M.S., Kaynak A., Kouzani A.Z. (2019). A Review on Miniaturized Ultrasonic Wireless Power Transfer to Implantable Medical Devices. IEEE Access..

[58] Sripadmanabhan Indira S., Aravind Vaithilingam C., Satya Prakash Oruganti K., Mohd F., Rahman S. (2019). Nanomaterials Nanogenerators as a Sustainable Power Source: State of Art, Applications, and Challenges. Nanomaterials.

[59] Wang X., Yang B., Liu J., Zhu Y., Yang C., He Q. (2016). A Flexible Triboelectric-Piezoelectric Hybrid Nanogenerator Based on P(VDF-TrFE) Nanofibers and PDMS/ MWCNT for Wearable Devices OPEN. Sci. Rep..

[60] Vidal J.V., Slabov V., Kholkin A.L., dos Santos M.P.S. (2021). Hybrid Triboelectric-Electromagnetic Nanogenerators for Mechanical Energy Harvesting: A Review. Nano-Micro Lett..

[61] Yu H., Zhou J., Yi X., Wu H., Wang W. (2015). A Hybrid Micro Vibration Energy Harvester with Power Management Circuit. Microelectron. Eng..

[62] Shao J., Willatzen M., Wang Z.L. (2020). Theoretical Modeling of Triboelectric Nanogenerators (TENGs). J. Appl. Phys..

[63] Niu S., Wang Z.L. (2015). Theoretical Systems of Triboelectric Nanogenerators. Nano Energy.

[64] Huang P., Wen D.-L., Qiu Y., Yang M.-H., Tu C., Zhong H.-S., Zhang X.-S. (2021). Micromachines Textile-Based Triboelectric Nanogenerators for Wearable Self-Powered Microsystems. Micromachines.

[65] Wang Z.L., Lin L., Chen J., Niu S., Zi Y. (2016). Triboelectric Nanogenerator: Single-Electrode Mode.

[66] Feng M., Wu Y., Feng Y., Dong Y., Liu Y., Peng J., Wang N., Xu S., Wang D. (2022). Highly Wearable, Machine-Washable, and Self-Cleaning Fabric-Based Triboelectric Nanogenerator for Wireless Drowning Sensors. Nano Energy.

[67] Suo G., Yu Y., Zhang Z., Wang S., Zhao P., Li J., Wang X. (2016). Piezoelectric and Triboelectric Dual Effects in Mechanical-Energy Harvesting Using BaTiO3 /Polydimethylsiloxane Composite Film. ACS Appl. Mater. Interfaces.

[68] Liu J., Yu D., Zheng Z., Huangfu G., Guo Y. (2021). Lead-Free BiFeO3 Film on Glass Fiber Fabric: Wearable Hybrid Piezoelectric-Triboelectric Nanogenerator. Ceram. Int..

[69] Han M., Zhang X., Liu W., Sun X., Peng X., Zhang H. (2013). Low-Frequency Wide-Band Hybrid Energy Harvester Based on Piezoelectric and Triboelectric Mechanism. Sci. China Technol. Sci..

[70] Cao L., Li Z., Guo C., Li P., Meng X., Wang T. (2019). Design and Test of the MEMS Coupled Piezoelectric–Electromagnetic Energy Harvester. Int. J. Precis. Eng. Manuf..

[71] Challa V.R., Prasad M.G., Fisher F.T. (2009). A Coupled Piezoelectric–Electromagnetic Energy Harvesting Technique for Achieving Power Output through Damping Matching. Smart Mater. Struct..

[72] Xia H., Chen R., Ren L. (2015). Analysis of Piezoelectric–Electromagnetic Hybrid Vibration Energy Harvester under Different Electrical Boundary Conditions. Sens. Actuators A Phys..

[73] Yu Z., Yang J., Cao J., Bian L., Li Z., Yuan X., Wang Z., Li Q., Dong S., Yu Z. (2022). A PMNN-PZT Piezoceramic Based Magneto-Mechano-Electric Coupled Energy Harvester. Adv. Funct. Mater..

[74] Shan X.B., Guan S.W., Liu Z.S., Xu Z.L., Xie T. (2013). A New Energy Harvester Using a Piezoelectric and Suspension Electromagnetic Mechanism. J. Zhejiang Univ. Sci. A.

[75] Yao M., Liu P., Wang H., Pham V.T. (2020). Nonlinear Dynamics and Power Generation on a New Bistable Piezoelectric-Electromagnetic Energy Harvester. Complexity.

[76] Shan X., Xu Z., Song R., Xie T. (2013). A New Mathematical Model for a Piezoelectric-Electromagnetic Hybrid Energy Harvester. Ferroelectrics.

